# Tumoricidal efficacy coincides with CD11c up-regulation in antigen-specific CD8^+^ T cells during vaccine immunotherapy

**DOI:** 10.1186/s13046-016-0416-x

**Published:** 2016-09-13

**Authors:** Yohei Takeda, Masahiro Azuma, Misako Matsumoto, Tsukasa Seya

**Affiliations:** Department of Microbiology and Immunology, Hokkaido University Graduate School of Medicine, Kita 15, Nishi 7, Kita-ku, Sapporo, 060-8638 Japan

**Keywords:** CTL, CD11c^+^ CD8^+^ T cell, Adjuvant, Poly(I:C), TLR3, Antitumor immunotherapy, Therapeutic marker

## Abstract

**Background:**

Dendritic cells (DCs) mount tumor-associated antigens (TAAs), and the double-stranded RNA adjuvant Poly(I:C) stimulates Toll-like receptor 3 (TLR3) signal in DC, which in turn induces type I interferon (IFN) and interleukin-12 (IL-12), then cross-primes cytotoxic T lymphocytes (CTLs). Proliferation of CTLs correlates with tumor regression. How these potent cells expand with high quality is crucial to the outcome of CTL therapy. However, good markers reflecting the efficacy of DC-target immunotherapy have not been addressed.

**Methods:**

Using an EG7 (ovalbumin, OVA-positive) tumor-implant mouse model, we examined what is a good marker for active CTL induction in treatment with Poly(I:C)/OVA.

**Results:**

Simultaneous administration of Poly(I:C) and antigen (Ag) OVA significantly increased a minor population of CD8^+^ T cells, that express CD11c in lymphoid and tumor sites. The numbers of the CD11c^+^ CD8^+^ T cells correlated with those of induced Ag-specific CD8^+^ T cells and tumor regression. The CD11c^+^ CD8^+^ T cell moiety was characterized by its high killing activity and IFN-γ-producing ability, which represent an active phenotype of the effector CTLs. Not only a TLR3-specific (TICAM-1-dependent) signal but also TLR2 (MyD88) signal in DC triggered the expansion of CD11c^+^ CD8^+^ T cells in tumor-bearing mice. Notably, human CD11c^+^ CD8^+^ T cells also proliferated in peripheral blood mononuclear cells (PBMC) stimulated with cytomegalovirus (CMV) Ag.

**Conclusions:**

CD11c expression in CD8^+^ T cells reflects anti-tumor CTL activity and would be a marker for immunotherapeutic efficacy in mouse models and probably cancer patients as well.

**Electronic supplementary material:**

The online version of this article (doi:10.1186/s13046-016-0416-x) contains supplementary material, which is available to authorized users.

## Background

Efficient induction of active CTLs is important for successful anti-tumor immunotherapy [1]. Poly(I:C), which is an adjuvant recognized by the pattern-recognition receptors (PRR) TLR3 and Melanoma differentiation-associated gene 5 (MDA5), acts on DCs to evoke potent CTL proliferation followed by tumor regression [2, 3]. There are three important steps for successful CTL-dependent tumor eradication: (i) CD8^+^ T cell priming in lymphoid organs; (ii) CTL infiltration into tumor sites; and (iii) keeping CTLs tumoricidal in tumor microenvironment. The first step, TAA and Poly(I:C) cross-prime Ag-specific CD8^+^ T cells by cross-presentation in DCs [4], which leads to CTL-dependent tumor regression [5, 6]. Poly(I:C) adjuvant accomplishes DCs maturation and the cross-presentation of exogenous Ags [7]. Poly(I:C) may also promote the second and third steps by acting on myeloid or stromal cells in lymph nodes and tumor.

Poly(I:C) facilitates the maturation of DCs and optimizes the Ag-presentation that culminates in the cross-presentation process. Additionally, Poly(I:C) up-regulates the expression of positive co-stimulatory molecules and the production of cytokines in mature DCs. The maturation of DC is also known to prime CD8^+^ T cells [7]. The second step concerns the migration of primed-CTLs to the tumor sites. Activated CD8^+^ T cells up-regulate expression of chemokine receptors such as CXCR3 and they are attracted to tumor sites expressing CXCR3 ligands such as CXCL9, CXCL10 and CXCL11 [8]. The third step concerns direct killing of tumor cells in the tumor sites. Tumors with poor prognosis frequently express the Programmed death-ligand 1 (PD-L1) protein, and this PD-L1-high environment inhibits CTL killing activity via the PD-L1/PD-1 signaling pathway [9, 10]. In mouse tumor models, the degree of tumor regression positively correlates with the expansion of tumor Ag-specific CD8^+^ T cells and the level of intratumor CD8^+^ T cells. These parameters are in turn influenced by the quality of DC maturation [9]. However, it is difficult to assess the therapeutic efficacy in anti-tumor immunotherapy by measuring such parameters in human cancer patients. Therefore, it is anticipated to search for simple markers measurable with patients’ blood that reflect the degrees of expansion and activation of Ag-specific CTLs.

In this study, we treated EG7 tumor-implant mice with OVA Ag and Poly(I:C): EG7 is an OVA-expressing tumor cell line, and able to assess TAA-specific CD8^+^ T cells by tetramer assay. We found that the activated CD8^+^ T cells expressed the CD11c molecule, and that most of the tumor-infiltrating CD8^+^ T cells were CD11c-positive in OVA and Poly(I:C)-treated mice. The up-regulation of CD11c expression on CD8^+^ T cells was evoked by various immunological stimuli, such as microbial infections or Ag and agonistic anti-4-1BB monoclonal antibody [11]. CD8^+^ T cells do not always have a uniform phenotype in terms of surface markers and functions. A bipolar immunological phenotype has been reported on the CD11c^+^ CD8^+^ T cell moieties: an effector function contributing to the elimination of pathogenic microbes and, controversially, a suppressive function reducing immune response [11].

Here we found that the Ag + Poly(I:C)-induced CD11c^+^ CD8^+^ T cells had an anti-tumor effector phenotype. We also observed that the degree of CD11c expression in intratumor CD8^+^ T cells paralleled their level of activation. The Ag-induced expansion of CD11c^+^ CD8^+^ T cells was also observed in human PBMCs. The level of CD11c expression in CD8^+^ T cells can be a useful marker for evaluation of the degree of expansion and the quality of tumor-specific CTLs as well as a marker to predict the efficacy of anti-tumor immunotherapies.

## Methods

### Mice

Wild-type C57BL/6 J mice were purchased from CLEA. *Ticam1−/−* and *Mavs−/−* mice were made in our laboratory. OT-1 mice were kindly provided by N. Ishii (Tohoku University, Miyagi, Japan). All mice were backcrossed >8 times to C57BL/6 background and maintained under specific pathogen-free condition in the animal faculty of the Hokkaido University Graduate School of Medicine. Animal experiments were performed according to the guidelines set by the animal safety center, Hokkaido University, Japan.

### Cell culture, reagents and antibodies

EL4 and EG7 cells were purchased from ATCC (VA, USA). WT1-C1498 cells were kindly provided by H. Sugiyama (Osaka University, Osaka, Japan) [12]. EL4 cells were cultured in RPMI 1640 (GIBCO, the catalog number: 11875-093, CA, USA) supplemented with 10 % heat-inactivated FBS (Thermo Fisher Scientific, SH30910.03, MA, USA) and 50 IU penicillin/50 μg/ml streptomycin (GIBCO, 15070-063). EG7 cells were cultured in RPMI 1640 supplemented with 10 % heat-inactivated FBS, 55 μM 2-mercaptoethanol (GIBCO, 21985-023), 10 mM HEPES (GIBCO, 15630-080), 1 mM sodium pyruvate (GIBCO, 11360-070), 50 IU penicillin/50 μg/ml streptomycin and 0.5 mg/ml G418 (Roche, 04 727 894 001, Basel, Schweiz). WT1-C1498 cells were cultured in RPMI 1640 supplemented with 10 % heat-inactivated FBS, 55 μM 2-mercaptoethanol, 50 IU penicillin/50 μg/ml streptomycin and 0.5 mg/ml G418. Poly(I:C) and MALP (macrophage-activating lipoprotein)-2 s were purchased from GE healthcare Life Sciences (the catalog number: 27-4732-01, IL, USA) and Biologica (Aichi, Japan), respectively. EndogGade® Ovalbumin (EndoOVA) was purchased from Hyglos (321001, Bayern, Germany). OVA257-264 peptide (SIINFEKL: SL8), OVA (H2Kb-SL8) Tetramer, WT1 (H-2Db-Db126) Tetramer, HLA-A*02:01 CMV pp65 Tetramer-NLVPMVATV-PE and HLA-A*24:02 CMV pp65 Tetramer-QYDPVAALF-PE were purchased from MBL (TS-5001-P, TS-5001-1, TS-M504-1, TS-0010-1C, TS-0020-1C, Aichi, Japan).

The following antibodies, anti-mouse CD3 (Clone: 145-2C11, the catalog number: 100306 and 100308), anti-mouse CD8α (53–6.7, 100729), anti-mouse CD11c (N418, 117317), anti-mouse CD16/32 (93, 101302), anti-mouse CD62L (MEL-14, 104405), anti-mouse CD103 (2E7, 121405), anti-mouse IFN-γ (XMG1.2, 505809), anti-mouse IL-2 (JES6-5H4), anti-mouse TNF-α (MP6-XT22, 506303), anti-human CD3 (HIT3a, 300317) and anti-human CD11c (3.9, 301613) were purchased from BioLegend (CA, USA). Anti-human CD8 (T8) was from BECKMAN COULTER (6603861, CA, USA).

Human FcR Blocking Reagent and CMV pp65-Recombinant Protein human Cytomegalovirus were purchased from Miltenyi Biotec (130-059-901, 130-091-824, Nordrhein-Westfalen, Germany). ViaProbe was purchased from BD Biosciences (555816, CA, USA). Chromium-51 Radionuclide was purchased from PerkinElmer (NEZ030S001MC, MA, USA).

### Reverse transcription-PCR and real-time PCR

In most samples, total RNA was prepared using TRIzol Reagent (Ambion, 15596018, TX, USA). Reverse transcription-PCR was carried out using a High Capacity cDNA Reverse Transcription kit (Applied Biosystems, 4368814, MA, USA). For total RNA purification from OVA-tetramer^+^ CD8^+^ T cells, CellAmp® Whole Transcriptome Amplification Kit (Real Time) Ver.2 (Takara, 3734, Shiga, Japan) was used according to the manufacturer’s instructions. Real-time PCR was performed using a Step One real-time PCR system (Applied Biosystems, 4368813). Sequences of primers in this study are shown in Additional file 1: Table S1. Levels of target mRNAs were normalized to *Gapdh* and fold-induction of transcripts was calculated using the ddCT method.

### Tumor challenge and adjuvant therapy

Mice were shaved at the back and subcutaneously injected with 200 μl of 2 × 10^6^ EG7 cells or 0.6 × 10^6^ WT1-C1498 cells in PBS. Tumor volume was calculated by using the formula: Tumor volume [mm^3^] = 0.52 × (long diameter [mm]) × (short diameter [mm]) ^2^. In the EG7 tumor bearing model, 100 μg of OVA with or without adjuvant (50 μg of Poly(I:C) or 50 nmol of MALP2s) was s.c. injected around tumor when the tumor volume reached about 200–600 mm^3^. OVA and adjuvant treatment was conducted once or twice at weekly intervals. 6 or 7 days after the last treatment, spleens, inguinal lymph nodes and tumor tissues were harvested for analysis. For measuring of intracellular IFN- γ, TNF-α and IL-2 staining, harvested cells were pulsed with 100 nM of SL8 for 6 h, and 10 μg/ml of Blefeldin A (Sigma-Aldrich, B7651-5MG, MO, USA) was added to the cells during the last 4 h. In WT1-C1498 tumor bearing model, PBS or 50 μg of Poly(I:C) was s.c. injected around tumor at day 5 and 12 after tumor implantation.

### Analysis of tumor-infiltrating lymphocytes

EG7 or WT1-C1498 tumors were excised from the tumor bearing mice. Isolated tumor was cut finely and treated with 0.05 mg/ml collagenase I (Sigma-Aldrich, C0130-100MG), 0.05 mg/ml collagenase IV (Sigma-Aldrich, C5138-1G), 0.025 mg/ml hyaluronidase (Sigma-Aldrich, H6254-500MG) and 0.01 mg/ml DNase I (Roche, 10 104 159 001) in Hank’s Balanced Salt Solution (Sigma-Aldrich, H9269-500ML) at 33 °C for 15 min. Tumor-infiltrating CD8^+^ T cells were analyzed and sorted on FACS AriaII (BD Biosciences).

### OT-1 proliferation assay

OT-1 T cells were prepared from spleens of OT-1 mice by CD8-microbeads (Miltenyi Biotec, 130-049-401). CD11c^+^ cells were isolated from spleens of wild-type mice by CD11c-microbeads (Miltenyi Biotec, 130-052-001). OT-1 T cells were labeled with 1 μM of CFSE for 10 min at 37 °C. 1 × 10^5^ CD11c^+^ cells and 2 × 10^4^ OT-1 T cells were co-cultured in 200 μl of medium with 4 μg/ml of OVA or OVA + 50 μg/ml of Poly(I:C) in 96-well flat bottom plate. After 60 h, these cells were stained with anti-CD8α, anti-CD3, and anti-CD11c. OT-1 T cell proliferation was determined with diminution of CFSE on FACS AriaII.

### Cytotoxic assay of CD8^+^ T cells

EG7 tumor bearing mice were challenged with OVA or OVA + Poly(I:C) on day 9 after tumor implantation. At day 15, CD8^+^ T cells were isolated from splenocytes by FACS sorting. EG7 and EL4 cells were labeled with ^51^Cr for 90 min and then washed three times with medium. CD8^+^ T cells (effecter cells) and ^51^Cr-labeled EG7 or EL4 (target cells) were co-cultured at the indicated ratio. After 4 h, supernatants were harvested and ^51^Cr release was measured in each sample. Specific lysis was calculated by using the formula: Cytotoxicity (%) = [(experimental release-spontaneous release) / (total release-spontaneous release)] × 100.

### *Ex vivo* CMV-specific CD8^+^ T cell immunization and HLA CMV tetramer assay

Human PBMCs were isolated from healthy donors by Ficoll-Paque PLUS (GE Healthcare Life sciences, 17-1440-02) following the manufacturer’s protocol. Haplotypes of donor’s HLA-A allele were checked by RapiType HLA-A for East Asian Pop (MBL, 4901). The donors’ HLA-A haplotypes included A*02 or A*24. The PBMCs were incubated with CMV pp65 protein in the presence or absence of 20 μg/ml of Poly(I:C) in RPMI 1640 medium supplemented with 5 % self plasma, 55 μM 2-mercaptoethanol, and 50 IU penicillin/50 μg/ml streptomycin. On day 2, equal volume of medium containing recombinant human IL-2 (Pepro Tech, AF-200-02-50UG, NJ, USA) was added to the culture dish. The final concentration of IL-2 was 50 U/ml. Two days later, the half volume of medium was changed to fresh medium containing IL-2 twice a week. After more than 8 days incubation, we analyzed the proportions of HLA CMV pp65-tetramer^+^ CD8^+^ T cells and CD11c^+^ CD8^+^ T cells on FACS AriaII.

### Statistical analysis

*P*-values were calculated with Kluskal-Wallis test with Dunn’s multiple comparison test or the Student’s *t*-test. Error bar represent the SD or SEM between samples.

## Results

### Antigen + Poly(I:C) induces the proliferation of CD11c^+^ CD8^+^ T cells in lymphoid tissue

**Fig. 1:**
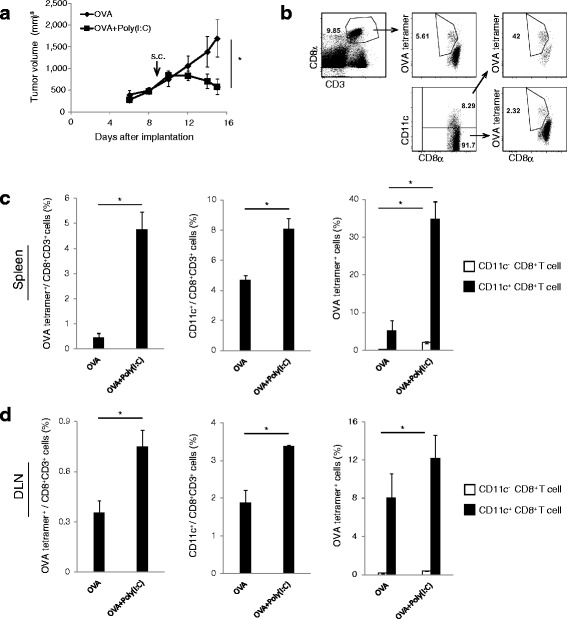
Antigen and Poly(I:C) administration increases the ratio of CD11c^+^ CD8^+^ T cells. **a** EG7 tumor was implanted to wild-type mice (C57BL/6 J), and OVA or OVA + Poly(I:C) was injected around tumor at day 9 after tumor implantation. Tumor volume was measured every 2 to 3 days. **b**–**d** 6 days after OVA administration, the proportions of OVA-specific CD8^+^ T cells and CD11c^+^ CD8^+^ T cells were evaluated on flow cytometer. In panel b, the gating strategy is shown. The proportions of each cell populations in spleen (c) and DLN (d) are calculated. Error bars show ± SEM; *n* = 4 to 5 per group. Student’s *t*-test was performed to analyze statistical significance. * *p* <0.05. The results are the representatives of three independent experiments

EG7 is an OVA-expressing lymphoma cell line (OVA positive-EL4 cell line) derived from the C57BL/6 mouse strain. The EG7 tumor is susceptible to CTL-mediated cytotoxicity and strongly suppressed by OVA and Poly(I:C) therapy [2]. We implanted EG7 in the back of C57BL/6 mice and administered OVA Ag alone or OVA + Poly(I:C) subcutaneously (s.c.) on day 9 after tumor implantation. The combination use of Poly(I:C) and OVA induced tumor regression in mice, whereas OVA alone treatment had only a minimal effect (Fig. 1a). On day 15, we harvested spleens and draining lymph nodes (DLN). The proportion of OVA-specific CD8^+^ T cells and CD11c^+^ CD8^+^ T cells were then analyzed (Fig. 1b). In both the spleen and DLN, OVA-specific CD8^+^ T cells and CD11c^+^ CD8^+^ T cells were increased in response to Poly(I:C) (Fig. 1c, d). Similar results were obtained with a non-tumor bearing model (Additional file 2: Figure S1a), thus CD11c^+^ population expanding as a result of the proliferation of OVA-specific CD8^+^ T cells in mice with OVA + Poly(I:C). The results indicate that CD11c^+^ CD8^+^ T cells contain the Ag-specific CD8^+^ T cells induced by Poly(I:C).

### Poly(I:C)-stimulated CD11c^+^ CD8^+^ T cells have a strong effector function

**Fig. 2:**
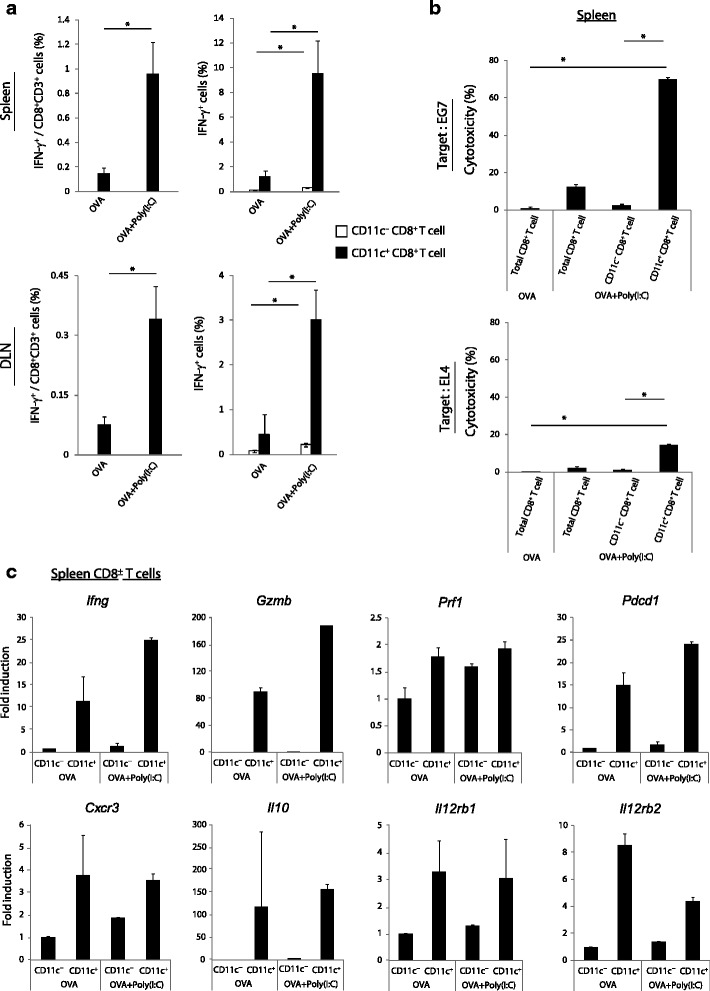
CD11c^+^ CD8^+^ T cells show anti-tumor phenotypes in response to Poly(I:C). **a** Mice loaded with EG7 tumor were treated with OVA or OVA + Poly(I:C) at day 9. Six days later, spleens and DLN were harvested and the proportions of IFN-γ^+^ CD8^+^ T cells were evaluated on flow cytometer. **b** CD11c^−^ CD8^+^ T cells and CD11c^+^ CD8^+^ T cells were isolated from splenocytes by FACS sorting at day 15. Isolated cells were co-cultured with ^51^Cr-laveled EG7 or EL4 (E/T = 40) for 4 h. Then, the cytotoxicity against EG7 and EL4 was measured by ^51^Cr-release assay. **c** CD11c^−^ CD8^+^ T cells and CD11c^+^ CD8^+^ T cells were isolated from spleens by FACS sorting at day 15. The gene expression levels were measured by quantitative PCR. Error bars show ± SEM; *n* = 4 to 5 per group (**a**) and ± SD (**b**, **c**). Student’s *t*-test (**a**) and Kluskal-Wallis test with Dunn’s multiple comparison test (**b**) were performed to analyze statistical significance. * *p* < 0.05. The results are one of the two independent experiments

We next assessed the phenotype of CD11c^+^ CD8^+^ T cells. In some foreign microbe infection models [13, 14] and a melanoma tumor-bearing model [15], the CD11c^+^ CD8^+^ T cell population reportedly expresses high IFN-γ-producing ability. In the EG7-bearing model, Poly(I:C) administration induced IFN-γ production by CD8^+^ T cells in the spleen and DLN (left in Fig. 2a). Within the Poly(I:C)-stimulated CD8^+^ T cell populations the CD11c^+^ population showed a higher ratio of IFN- γ^+^ cells compared to the CD11c- population (right in Fig. 2a). Similar results were obtained with cells from a non-tumor bearing mouse model (Additional file 2: Figure S1b).

Next, we measured the cytotoxic potential of the Poly(I:C)-stimulated CD11c^+^ CD8^+^ T cells in the spleen. Total CD8^+^ T cells and the CD11c- and CD11c^+^ populations were isolated from the spleens of OVA + Poly(I:C)-treated EG7-tumor bearing mice and co-cultured with ^51^Cr-labeled EG7 or EL4 cells. EL4 represents the basal cell line with no OVA Ag. The level of CD8^+^ T cell-induced cytotoxicity was measured using a ^51^Cr-release assay. Total CD8^+^ T cells isolated from mice which had been treated with OVA alone were the negative control. Poly(I:C)-stimulated CD11c^+^ cells showed markedly higher cytotoxicity towards EG7 cells (top in Fig. 2b) and EL4 cells (bottom in Fig. 2b) compared with other groups. Similar results were observed with the OVA + Poly(I:C)-immunized non-tumor bearing model (Additional file 2: Figure S1c). Hence in this model, the OVA + Poly(I:C)-stimulated CD11c^+^ population attacked mainly OVA-positive EG7 cells and reactively OVA-negative EL4 cells. These results indicate that the Poly(I:C)-stimulated CD11c^+^ cells exerts OVA-specific, as well as weak by-standing cytotoxicity toward basal EL4 Ag.

Microarray analysis with CD11c^+^ CD8^+^ T cells increased in malaria parasite vaccination exhibits the differential gene-inducing program compared to CD11c^−^ CD8^+^ T cells [14]. Therefore, we analyzed the gene expression profiles of CD11c^−^ and CD11c^+^ populations isolated from the spleens of tumor-bearing mice in the presence or absence of Poly(I:C) stimulation (Fig. 2c). Genes such as *Ifng*, *Gzmb* (which indicate the CTL effector function) and *Il12r*, were highly expressed in the CD11c^+^ population compared to the CD11c^−^ population in both OVA alone and OVA + Poly(I:C) groups. *Cxcr3*, which is a chemokine receptor involved in T cell migration, was also higher in the CD11c^+^ cells. Although CD11c^+^ cells showed high effector gene expression patterns, *Pdcd1* (PD1 gene) and *Il10* expression was also high. When comparing the group treated with OVA alone to that treated with OVA + Poly(I:C), the expression levels of *Ifng*, *Gzmb* and *Pdcd1* in the CD11c^+^ cells were up-regulated in response to Poly(I:C) stimulation.

Subsequently, we checked the expression of other molecules which have been reported as the activated- and effector-CTL markers (Additional file 3: Figure S2). The down-regulation of CD62L is one of the effector markers for CD8^+^ T cells [16] and the splenic CD11c^+^ CD8^+^ T cells showed higher ratio of CD62L^−^ phenotype than CD11c^−^ cells. PD-1 expression was consistent with the gene (*Pdcd1*) expression. In Malaria infection-mouse models, CD11c^+^ CD8^+^ T cells showed a KLRG^+^ CD127^−^ pattern, which is known as a marker of short-lived effector cells [14]. In the present EG7-bearing models, CD11^+^ populations showed a similar pattern. Since the high IFN-γ-producing ability and anti-tumor ability of CD103^+^ CD8^+^ T cells were reported [17, 18], we checked the levels of CD103 expression. However, no positive correlation between CD11c and CD103 expression on CD8^+^ T cells was observed (Additional file 3: Figure S2a). The TNF-α and IL-2-producing ability of CD11c^+^ population was much higher than CD11c- population (Additional file 3: Figure S2b). We further analyzed the gene expression pattern in splenic CD11c^+^ and CD11c^−^ populations (top in Additional file 3: Figure S2c). CD11c^+^ cells showed higher *Klrg1* expression than CD11c^−^ cells, and the expression level was further increased by Poly(I:C)-stimulation. *Cd127* and *Cd103* expression levels were lower in CD11c^+^ cells than CD11c^−^ cells in both of OVA and OVA + Poly(I:C) groups. We additionally checked the markers of CD8^+^ Tregs: *Tgfb* and *Cd122* [19], and CD11c^+^ cells only modestly expressed these genes. Thus, the CD11c^+^ expression represents a short-lived effector phenotype of CD8^+^ T cells.

### Intratumor antigen-specific CD11c^+^ CD8^+^ T cells demonstrate the effector phenotype

**Fig. 3:**
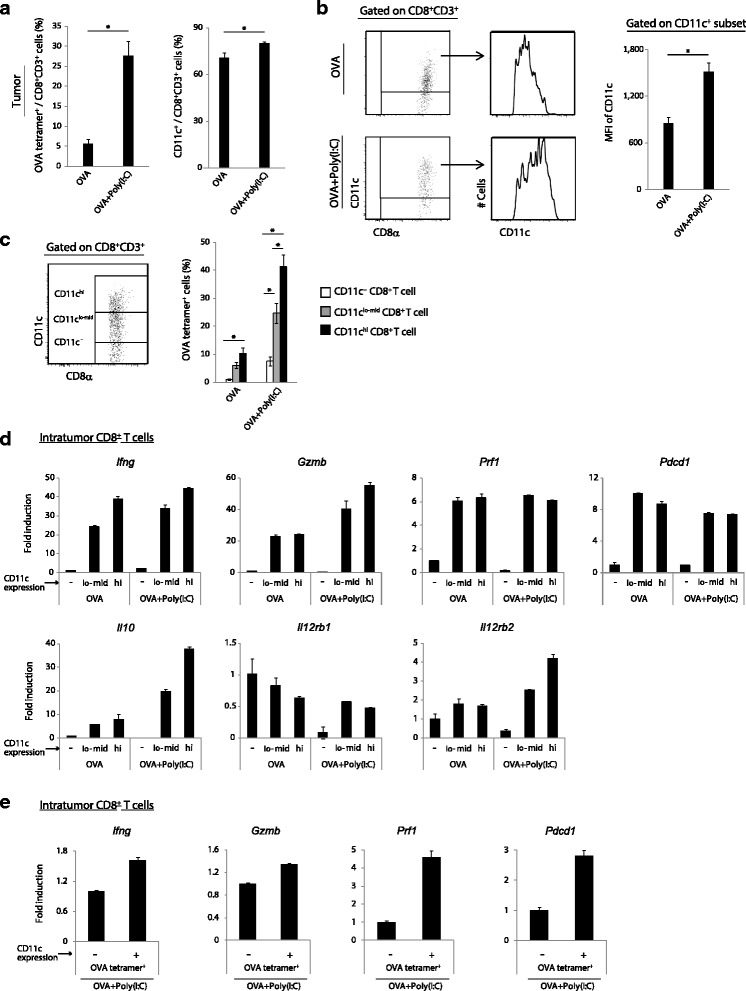
CD11c expression is correlated with activation of tumor-infiltrating CD8^+^ T cells. **a** EG7 tumor bearing mice were treated with OVA or OVA + Poly(I:C) at day 9. Six days later, tumors were harvested and the proportions of OVA-specific CD8^+^ T cells and CD11c^+^ CD8^+^ T cells were evaluated on flow cytometer. **b** CD11c expression level on intratumor CD8^+^ T cells was evaluated on flow cytometer. Gating strategy is shown in the left panels, and the CD11c expression levels on CD11c^+^ CD8^+^ T cells are shown in the right panel. **c** The proportions of OVA-specific populations on intratumor CD11c^−^ , CD11c^lo-mid^ and CD11c^hi^ CD8^+^ T cells were evaluated on flow cytometer. Gating strategy is shown in the left panel. Error bars show ± SEM; *n* = 5 per group. Student’s *t*-test was performed to analyze statistical significance. * *p* < 0.05(**a**-**c**). **d**-**e** CD11c^−^ , CD11c^lo-mid^ and CD11c^hi^ CD8^+^ T cells (**d**) or OVA + Poly(I:C)-stimulated OVA-tetramer^+^ CD11c^−^ and CD11c^+^ CD8^+^ T cells (**e**) were isolated from tumor tissues by FACS sorting at day 15. The gene expression levels were measured by quantitative PCR. Error bars show ± SD (**d**, **e**). The results are one of the two independent experiments

The infiltration of CTL into tumor and maintenance of their cytotoxicity are crucial for successful anti-tumor immunotherapy [9, 20]. Then, we assessed the properties of intratumor CD8^+^ T cell populations. First, we measured the proportions of intratumor OVA-specific CD8^+^ T cells and CD11c^+^ CD8^+^ T cells in tumor of EG7-bearing mice treated with OVA alone and OVA + Poly(I:C). The expansion of intratumor OVA-specific CD8^+^ T cells was strongly induced following OVA + Poly(I:C) administration (left in Fig. 3a). Approximately 70 % of intratumor CD8^+^ T cells expressed the CD11c molecule even in the absence of Poly(I:C) stimulation, and the ratio was further increased in the CD11c + population by Poly(I:C) (right in Fig. 3a). In addition to the expansion of the CD11c^+^ moiety, Poly(I:C) up-regulated the expression level of CD11c on the CD11c^+^ T cell population (Fig. 3b).

In the OVA + Poly(I:C) group we focused on two representative mice with different responses: one with a high degree of tumor regression (the tumor volume at day 12 was ≤ 200 mm^3^) and the other with a moderate degree of regression (the tumor volume at day 12 was ≥ 200 mm^3^) (Additional file 4: Figure S3a). The ratios of intratumor OVA-specific and CD11c^+^ populations in CD8^+^ T cells were markedly high in the mice with mild-regressing tumor, but low in the mice with fast-regressing tumor (Additional file 4: Figure S3b). Hence, OVA-specific CD11c^+^ CD8^+^ T cells are efficiently expanded and enter into the tumor during tumor regression and then disappear prior to remission.

Next, we assessed the parallelism between the CD11c expression level and the ratio of OVA-specific T cells. They appeared to be positively correlated (Fig. 3c); hence, we separated CD11c^−^ , CD11c^lo-mid^ and CD11c^hi^ subpopulations from tumor tissue and analyzed their markers. The expression levels of *Ifng*, *Gzmb*, *Il10* and *Il12rb2* were higher on the CD11c^hi^ than the CD11c^lo-mid^ subpopulation in the OVA + Poly(I:C) group (Fig. 3d). These results indicate that the degree of CD11c expression in intratumor CD8^+^ T cells reflects the intense activation of Ag-specific CD8^+^ T cells. The gene expression patterns between CD11c^−^ and CD11c^+^ populations in OVA-specific CD8^+^ T cells showed that the levels of *Ifng*, *Gzmb*, *Prf1* and *Pdcd1* were higher in the OVA-tetramer^+^ CD11c^+^ cells than in OVA-tetramer^+^ CD11c^−^ cells (Fig. 3e). The expression pattern of other genes: *Cd127*, *Klrg1*, *Cd103*, *Tgfb2* and *Cd122*, in intratumor CD11c^−^ and CD11c^+^ cells was almost consistent with those in splenic CD11c^−^ and CD11c^+^ CD8^+^ T cells (Additional file 3: Figure S2c). We further assessed the gene expression levels of exhaustion markers such as *Tim3* and *Lag3* in intratumor CD11c^−^ and CD11c^+^ CD8^+^ T cells [10]. *Tim3* expression levels of intratumor CD11c^−^ and CD11c^+^ cells were very low and *Lag3* expression was unchanged by Poly(I:C) stimulation in both CD11c^−^ and CD11c^+^ cells (Additional file 3: Figure S2c). These results indicate that intratumor CD11c^+^ CD8^+^ T cells are non-exhausted/short-lived effector CD8^+^ T cells. Possibly, up-regulation of the CD11c and activation of CD8^+^ T cells may coincidentally occurs in response to Poly(I:C) at the CD8^+^ T cell-priming stage.

### The level of CD11c expression correlates with the degree of Ag-specific cell division

**Fig. 4:**
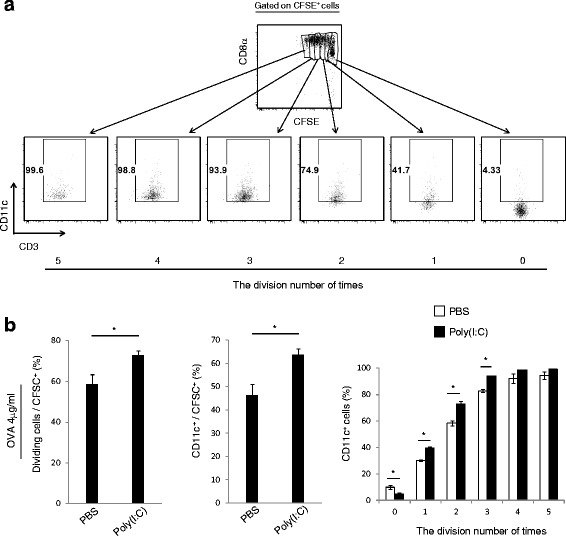
Antigen-specific cell proliferation correlates with CD11c expression in CD8^+^ T cells. **a** CD11c^+^ DCs were isolated from spleens of wild-type mice and incubated with OVA in the presence or absence of Poly(I:C) for 4 h, and then co-cultured with CFSE-labeled OT-1 T cells. After 60 h, antigen-specific proliferation was evaluated by diminution of CFSE with reference to cells gated. **b** The cycles of cell division and levels of CD11c^+^ OT-1 T cells are shown (the left and the middle panel). Division times and CD11c^+^ levels are analyzed with samples in panel A (the right panel). Error bars show ± SEM. Student’s *t*-test was performed to analyze statistical significance. * *p* < 0.05. The results are the representatives of three independent experiments

**Fig. 5:**
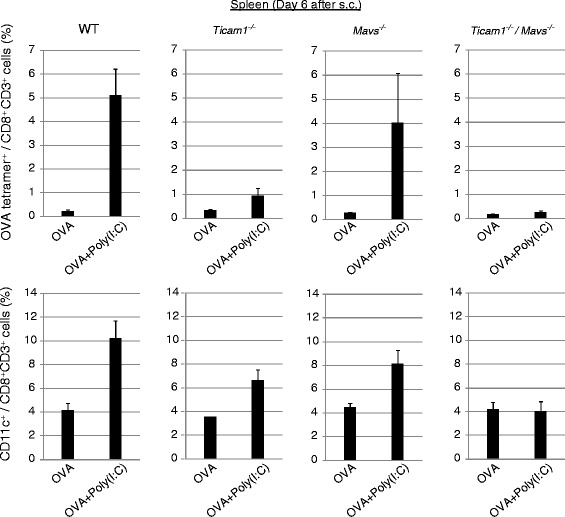
Poly(I:C) increases CD11c^+^ CD8^+^ T cells independent of TICAM-1 and MAVS. KO mice were challenged with EG7 cells, and treated with OVA with or without Poly(I:C) at day 9. Six days after OVA administration, spleens were harvested and the proportions of OVA-specific CD8^+^ T cells and CD11c^+^ CD8^+^ T cells were evaluated on flow cytometer. Error bars show ± SEM; *n* = 2 to 3 per group

To validate the above hypothesis, an in vitro OT-1 proliferation assay was performed. CFSE-labeled OT-1 cells were co-cultured with CD11c^+^ DCs in the presence of OVA, with or without Poly(I:C) stimulation. Sixty hours after co-culture the correlation between the number of cell divisions and the ratio of different OT-1 CD11^+^ cells was assessed by FACS gating (Fig. 4a). The rate of OT-1 proliferation and proportion of CD11c^+^ cells was increased by Poly(I:C) stimulation (left and middle in Fig. 4b). Furthermore, the proportion of CD11c^+^ cells gradually increased in proportion to the number of cell division, and almost all cells that divided more than three times were CD11c-positive (right in Fig. 4b). The CD11c expression was highly induced in the Poly(I:C) group, but this stimulation was just additive in this in vitro model. The results indicate that if the priming signal of CD8^+^ T cell is sufficiently robust, no specific second stimulation is mandatory to induce CD11c^+^ expression; similar results were obtained using an in vivo tetramer assay (Fig. 5). In this assay, *Ticam1*, *Mavs* and *Ticam1/Mavs* knockout mice were immunized with OVA + Poly(I:C) and the ratios of OVA-specific CD8^+^ T cells and CD11c^+^ CD8^+^ T cells were measured. The level of induction of CD11c^+^ cells paralleled the number of OVA-specific CD8^+^ T cells (Fig. 5).

We next confirmed this using another adjuvant. Wild-type mice were immunized with OVA + MALP2s [21], which is a TLR2 agonist that induces DC maturation using a different mechanism from Poly(I:C) (Additional file 5: Figure S4, exact differences will be shown elsewhere). Like OVA + Poly(I:C), OVA + MALP2s therapy also induced EG7 tumor regression (Additional file 5: Figure S4a). Further analysis revealed that MALP2s up-regulated the proportion of OVA-specific CD8^+^ and CD11c^+^ CD8^+^ T cells, as with Poly(I:C) treatment (Additional file 5: Figure S4b). Hence, it is likely that the occurrence of the CD11c^+^ population relies not only on the TICAM-1 pathway but the MyD88 pathway which up-regulate the cross-presentation.

### Poly(I:C) therapy induces CD11c^+^ CD8^+^ T cells in the WT1-expressing tumor-bearing model

**Fig. 6:**
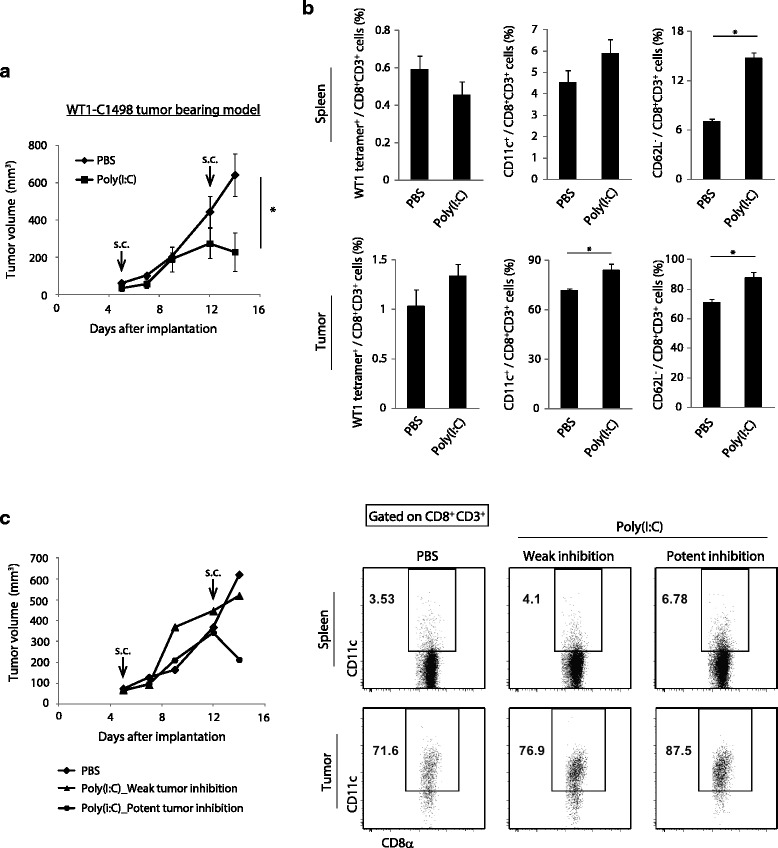
Poly(I:C) therapy induces the expansion of CD11c^+^ CD8^+^ T cells in a WT1 tumor model. **a** Wild-type mice challenged with WT1-C1498 tumor cells were treated with PBS or Poly(I:C) around tumor at day 5 and 12 after implantation. Tumor volume was measured every 2 to 3 days. **b** At day 14, spleen and tumor were harvested and the proportions of WT1-specific CD8^+^ T cells, CD11c^+^ CD8^+^ T cells and CD62L^−^ CD8^+^ T cells were evaluated on flow cytometer. **c** The representative mice were selected from the PBS and Poly(I:C) group. Tumor growth and the proportions of CD11c^+^ CD8^+^ T cells in spleen and tumor are shown. In the left panel, diamond indicates PBS-treated control. triangle indicates Poly(I:C) low-responding mice. Circle indicates Poly(I:C) high-responding ones. Error bars show ± SEM; *n* = 3 to 4 per group. Student’s *t*-test was performed to analyze statistical significance. * *p* < 0.05. The results are one of the two independent experiments

We next assessed the correlation between Poly(I:C) efficacy and CD11c^+^ CD8^+^ T cell proliferation in WT1-C1498, a WT1-expressing leukemia cell line derived from the C57BL/6 mouse strain that is known to be sensitive to CTL-mediated cytotoxicity [12]. The Wilms’ tumor gene, *WT1*, is expressed in several human hematological malignancies and solid tumors [22]. The WT1 protein has immunogenicity and hence the potential to induce T cell activation [22]. We implanted WT1-C1498 cells into mice and subcutaneously injected PBS or Poly(I:C) to the mice at days 5 and 12 after tumor cell implantation. The Poly(I:C) treatment lead to retardation of tumor growth (Fig. 6a). In this model, WT1 protein was not added to Poly(I:C), and WT1-specific CD8^+^ T cells was only subtly induced; the Poly(I:C)-induced increase in WT1-tetramer^+^ CD8^+^ T cells was below the detection limit in spleen and tumor tissue (Fig. 6b). However, the proportion of CD62L-negative CD8^+^ T cells was increased following Poly(I:C) treatment (Fig. 6b). This result indicates that activation of CD8^+^ T cells is induced by Poly(I:C) monotherapy in WT1-expressing tumors.

In this context, the proportion of CD11c^+^ CD8^+^ T cells was also increased following Poly(I:C) administration (Fig. 6b). The intratumor CD11c^+^ CD8^+^ T cells showed higher cytotoxic genes expression than CD11c^−^ CD8^+^ T cells in PBS-control mice. These genes expression in CD11c^+^ CD8^+^ T cells were increased more by Poly(I:C) treatment as in EG7-bearing mice (Additional file 6: Figure S5). Although Poly(I:C)-treatment induced tumor growth retardation, the degree of the growth inhibitionappeared to be different among individual mice. (left in Fig. 6c). Further analysis revealed that the expansion of CD11c^+^ CD8^+^ T cells was minimal in the former, while robustly induced in the latter (right in Fig. 6c). Anyhow, CD11c^+^ CD8^+^ T cells proliferate in tumor in response to Poly(I:C) irrespective of tumor types.

### Antigen immunization induces CD11c^+^ CD8^+^ T cell expansion in human PBMCs

**Fig. 7:**
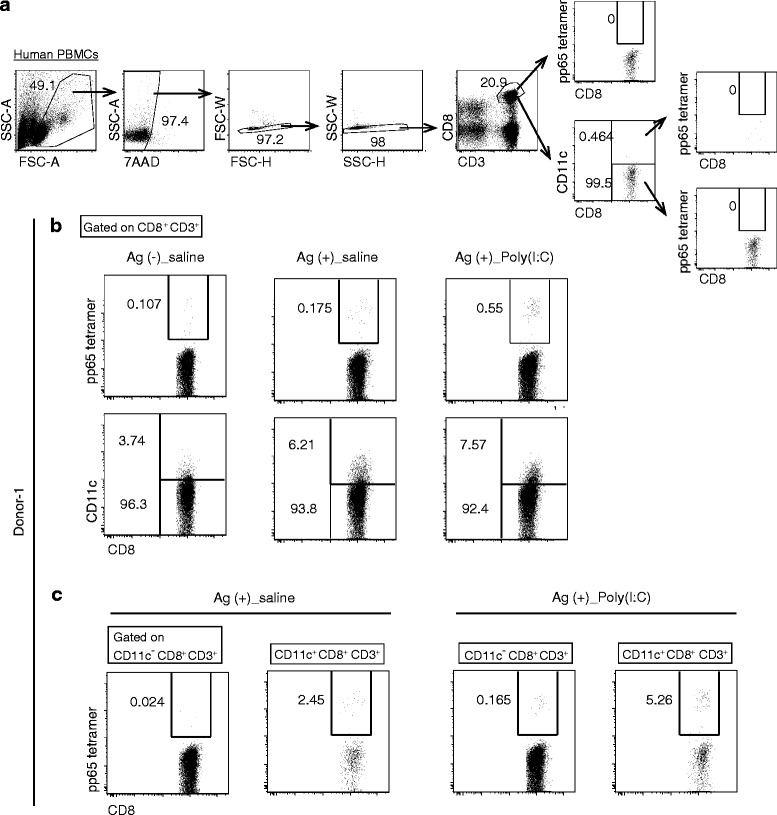
CD11c^+^ CD8^+^ T cells expand in response to Ag immunization in human PBMCs. Human PBMCs isolated from donor-1 were treated with CMV pp65 protein in the presence or absence of Poly(I:C). Negative control was a sample without Ag and Poly(I:C). Eight days after the treatment, the proportions of CMV pp65 tetramer^+^ CD8^+^ T cells and CD11c^+^ CD8^+^ T cells were evaluated on flow cytometer. **a** The gating strategy is shown. Samples were labeled with 7AAD, α-CD8 Ab, α-CD3 Ab or isotype of α-CD11c Ab without tetramer staining. **b** Upper panels show the percentage of pp65 tetramer^+^ cells in CD8^+^ T cells. Lower panels show the percentage of CD11c^+^ cells in CD8^+^ T cells. **c** The percentage of pp65 tetramer^+^ cells in CD11c negative- and CD11c positive-CD8^+^ T cells are shown

Finally, we tested whether CD11c^+^ CD8^+^ T cells proliferate in a human in vitro system. PBMCs were isolated from four healthy donors and immunized with CMV pp65 protein, in the presence or absence of Poly(I:C) stimulation. After more than 8 days incubation, the proportions of CMV-tetramer^+^ and CD11c^+^ CD8^+^ T cells were analyzed (Fig. 7a). Regarding Donor-1-derived PBMCs, CMV-tetramer^+^ CD8^+^ T cells were barely detectable in Ag-free conditions and approximately 4 % of CD8^+^ T cells expressed CD11c (left panels in Fig. 7b). Under Ag-positive, Poly(I:C)-free conditions, CMV-tetramer^+^ CD8^+^ T cells increased slightly and the percentage of CD11c^+^ cells increased to more than 6 % (middle panels in Fig. 7b). When Poly(I:C) stimulation was accompanied by Ag immunization, the expansion of CMV-tetramer^+^ CD8^+^ T cells was induced more potently and CD11c^+^ cells reached around 7.5 % (right panels in Fig. 7b). In these Ag-positive situations, CD11c^+^ population contained more Ag-specific CD8^+^ T cells than CD11c^−^ ones (Fig. 7c). In PBMCs isolated from other donors, CMV-tetramer^+^ CD8^+^ T cells and CD11c^+^ CD8^+^ T cells were increased with Ag-only stimulation (Additional file 7: Figure S6), which is consistent with the result of Donor-1. Yet no additive increase was observed by Poly(I:C) in this setting (data not shown). Upon Ag + Poly(I:C)-stimulation in PBMC samples, the dynamics of tetramer^+^ CD8^+^ T cells and CD11c^+^ CD8^+^ T cells were individually diverged, suggesting the limit of the in vitro assay for Poly(I:C) function in some human samples. We would say, at least CD11c^+^ CD8^+^ T cells are increased with Ag stimulation and the levels clearly correlate with those of Ag-specific CD8^+^ T cells in human immune cells, partly as per the mouse models.

## Discussion

In the present study of anti-tumor vaccine therapy, we found that CD8^+^ T cells primed by DCs express the CD11c molecule, which parallels the intensity of the proliferation signal in the CD8^+^ T cells. We also demonstrated that primed CD11c^+^ CD8^+^ T cells exert an effector phenotype and contribute to tumor regression in mouse; in two tumor-implant models, EG7(OVA) and C1498(WT1), CD11c^+^ CD8^+^ T cell expansion is feasible accompanied with potent therapeutic efficacy. CD11c reflects an activation state of CD8^+^ T cells with killing activity in inflammatory microenvironment, encompassing infection and tumor.

CD11c is a subunit of beta2 integrin, namely p150,95 or CR4 [23] and selectively expressed on myeloid cells, in particular DCs [24]. The molecule is also expressed on a minor population of CD8^+^ T cells in spleen, lymph node, thymus, liver and bone marrow in naïve mouse models [25, 26]. CD11c^+^ CD8^+^ T cells also exist in the intestines where the proportion of CD11c^+^ cells is higher than other organs [25, 27]. When mice are infected with pathogenic microbes such as bacteria, protozoa and viruses, the CD11c^+^ T cell moiety is dramatically expanded in lymphoid tissues, blood and infected lesions [14, 28, 29]. In infection models, the CD11c^+^ T cells have been shown to exhibit a short-lived effecter phenotype, with high killing activity and IFN-γ-producing ability [13, 14, 28, 29]. In a *Listeria monocytogenes*-infected mouse model, the killing ability of CD11c^+^ CD8^+^ T cells is reduced by the blocking of CD11c [13], which illustrates that CD11c is not only a phenotypic marker of activated CD8^+^ T cells, but also plays an important role in mediating their cytotoxic effect. Since CD11c is a receptor for C3bi [23, 30], it enhances interaction with C3bi-coated particle [31]. Tumor cells tend to be labeled with C3bi in inflammatory environment, which might promote CTL-mediated tumor cell clearance [32].

Based on earlier reports on a increase of the CD11c^+^ CD8^+^ T population in infections [13, 14, 28, 29], there might be two possibilities in expansion of CD11c population in CD8^+^ T cells: inducible of CD11c in popular CD8^+^ T cells or proliferation of a unique CD8^+^ T subset that essentially expresses CD11c. In our in vivo setting with tumor-infiltrated CD8^+^ T cells, the CD11c level in CD8^+^ T cells is up-regulated in accordance with number of the CD11c^+^ CD8^+^ T cells in response to Poly(I:C). Thus, the both mechanisms appear to participate in abundance of CD11c-positive cells in CD8^+^ T cell population in tumor when the mice are stimulated with Poly(I:C). In a resting state of mouse, there exists a minor population of CD11c^+^ CD8^+^ T cells, which needs to be further analyzed.

We previously showed that the single administration of OVA failed to induce OVA-specific CD8^+^ T cell proliferation in spleen [3]. However, a small number of CD11c^+^ CD8^+^ T cells exist in both the tumor-bearing and non-tumor-bearing models, even in the absence of Poly(I:C) stimulation (Fig. 1c and Additional file 2: Figure S1a). The constitutive CD11c^+^ T cells in spleen demonstrate a similar gene expression profile as effector CD8^+^ T cells (Fig. 2c). Only a few CD8^+^ T cells expressing CD11c would remain active in a non-stimulated state. The homeostatic activation of CD11c^+^ CD8^+^ T cells may include putative Ag-specific memory CD8^+^ T cells recognizing tumor-associated or self-Ags. Although these activated T cells are numerically and qualitatively insufficient to induce tumor regression, the CD11c^+^ CD8^+^ T cell population expands to enhance the effector function once Poly(I:C) stimulation primes Ag-specific CD8^+^ T cells in mice.

In contrast to the activation of CD8^+^ T cells [13, 28], several papers have focused on the suppressive functions of the CD11c^+^ CD8^+^ T cells. The CD11c^hi^ population is able to kill activated CD4^+^ T cells in *Listeria*-infected mice [13]. In 4-1BB-stimulated rheumatoid arthritis or autoimmune uveoretinitis mouse models, IFN-γ produced from CD11c^+^ CD8^+^ T cells up-regulates. Indoleamine-pyrrole 2,3-dioxygenase (IDO) enzyme in DCs and macrophages, which ameliorates inflammatory-mediated tissue damage [33, 34]. Thus, some of the immunosuppressive functions of CD11c^+^ CD8^+^ T cells occur in tumor secondary to their strong effector function, to prevent excessive or abrupt tumor destruction.

Regarding intratumor CD11c^+^ CD8^+^ T cells, their number and phenotype reflect both the migration and effector phases. The proportion of the CD11c^+^ population in CD8^+^ T cells is much higher in tumor than in spleen or DLN (Figs. 1c, d and 3a), with over 60 % of the intratumor CD8^+^ T cells expressing the CD11c molecule. The same distribution is observed in B16-F10 melanoma-bearing mouse models given the agonistic anti-4-1BB monoclonal antibody-monotherapy or combination therapy with anti-CD4 antibody [15, 35]. The primed CD8^+^ T cells acquire the ability to migrate, presumably through the up-regulation of the chemokine receptor Cxcr3 in spleen; thus these primed CD11c^+^ CD8^+^ T cells may preferentially accumulate in tumor bed. When CD8^+^ T cells are activated, the level of PD-1 expression is up-regulated via T cell receptor signaling [36]. These activated CTLs may exhibit high tumoricidal activity but exhaust easily in a high PD-L1 tumor microenvironment. Hence, the number and activation state of CD8^+^ T cells infiltrating into the tumor would be in temporally parallel. In our EG7-bearing model, intratumor CD11c^+^ cells showed similar gene expression profiles to splenic CD11c^+^ CD8^+^ T cells (Fig. 3d, e) with the properties of active CTL on the way to tumor regression. CD11c CD8^+^ T cells may be increased as an activation marker in human PBMC samples stimulated with Ag. Therefore, as a prediction marker for therapeutic efficacy in patients, the number and level of CD11c expression in CD11c^+^ CD8^+^ T cells in blood would predict the quality of intratumor CTLs in cancer patients having immunotherapy.

In clinical human cancer immunotherapy, short peptides containing epitopes are found apparently ineffective [37, 38]. The main problems of short peptides therapy include low immunogenicity and HLA-restriction [39]. Whole tumor-Ag protein is difficult to provide because of the difficulty in synthesizing high-grade protein [18]. Poly(I:C)-monotherapy is more practical than treatment with TAA + Poly(I:C) in patients with cancer having unidentified TAA as per BCG-CWS thrapy [40, 41]. However, the Poly(I:C)-monotherapy appears insufficient to support the potent priming of Ag-specific CD8^+^ T cells compared with Ag + Poly(I:C) therapy, as shown in C1498 (WT1) versus EG7 (OVA)-bearing mouse models [2, 3, 42]. In addition, there appears to be an individual-to-individual difference in the response to Poly(I:C) treatment (Fig. 7 and Additional file 7: Figure S6). Expansion of CD11c^+^ CD8^+^ T cells should be judged based on the reflection of tumor regression and therapeutic efficacy.

Finally, we used an *ex vivo* HLA CMV-tetramer assay for testing expansion of Ag-specific CD11c^+^ T cells in human PBMC samples. Results showed an obvious expansion of CD11c^+^ population following the immunization by CMV Ag in Poly(I:C)-free conditions (Fig. 7). However, this tendency of tetramer^+^ and CD11c^+^ CD8^+^ T cells was poorly reproduced in some other donors in response to Ag + Poly(I:C)-stimulation. Since Poly(I:C) induces activation-induced or necroptotic cell death in various types of cells [43, 44], the Poly(I:C) dose may have critically affected the PBMC conditions in this artificial assay system [45]. Yet, these human PBMC studies suggest that Ag-stimulation impacts the link between activation and increase of CD11c^+^ CD8^+^ T cells as in the mouse models.

## Conclusions

In conclusion, CD11c^+^ CD8^+^ T cell population crucially expands in anti-tumor immunotherapy, and reflects proliferation of tumor antigen-specific CTL in mouse tumor-implant models. Expansion of CD11c^+^ CD8^+^ T cell population reveals a possible marker for successful immunotherapy in mouse, and possibly in human clinical tests. It would be more suitable and convenient to evaluate overall CD8^+^ T expansion and CD11c marker than to employ a tetramer assay with unidentified Ags, but we need to check the period when this parallelism is observed. The expansion of CD11c^+^ CD8^+^ T cells in response to vaccine and adjuvant would be a simple and practical marker for the adaptability of immunotherapy to cancer patients.

## Additional files

Additional file 1: Table S1. Primer sequences used for real-time RT-PCR. (DOCX 20.7 kb) Additional file 2: Figure S1. CD11c^+^ CD8^+^ T cells show Ag + Poly(I:C)-induced effector functions in non-tumor bearing mice. a-b OVA with or without Poly(I:C) was s.c. injected to tumor-unloading mice. Seven days later, spleens and DLN were harvested and the proportions of OVA-specific CD8^+^ T cells, CD11c^+^ CD8^+^ T cells (a) and IFN-g^+^ CD8^+^ T cells (b) were evaluated by flow cytometer. Error bars show ± SEM; n = 5 per group. Student’s *t*-test was performed for statistical significance. * *p* < 0.05 (a, b). c CD11c- CD8^+^ T cells and CD11c^+^ CD8^+^ T cells were isolated from spleens by flow cytometer sorting at day 7. Isolated cells were cocultured with ^51^Cr-labeled EG7 or EL4 (E/T = 20) for 4 hours. Then, the cytotoxicity against EG7 and EL4 was measured by ^51^Cr-release assay. Error bars show ± SEM. (DOCX 128 kb) Additional file 3: Figure S2. Gene expression profile of CD11c^+^ CD8^+^ T cells. a-b EG7 tumor-bearing mice were treated with OVA or OVA + Poly(I:C) at day 9. Six days later, spleens were harvested and the proportions of CD62L^-^, PD-1^+^, KLRG1^+^ /CD127^-^ and CD103^+^ of the CD8^+^ T cells (a) and TNF-a^+^ and IL-2^+^ of the CD8^+^ T cells (b) were evaluated by flow cytometer. c CD11c^-^ CD8^+^ and CD11c^+^ CD8^+^ T cells were isolated from spleens and tumors by FACS sorting at day 15. The gene expression levels were measured by quantitative PCR. Error bars show ± SEM; n = 3 to 9 per group (a, b) and ± SD (c). Student’s *t*-test was performed for statistical significance. * p < 0.05. The results are one of the two independent experiments. (DOCX 214 kb) Additional file 4: Figure S3. Tumor-infiltrating CD11c^+^ CD8^+^ T cells disappear immediately before the complete tumor regression. a EG7 tumor-bearing mice were administered with OVA and Poly(I:C) around tumor at day 5 after tumor implantation. Tumor volume was measured every 2 to 3 days. Tumors with mild (+) and rapid (++) regression profiles (≥ 200 mm^3^ at day 12 vs. ≤ 200 mm^3^ at day 12) were used in this study. b 7 days after OVA treatment (on day 12), the proportions of intratumor OVA-specific CD8^+^ T cells and CD11c^+^ CD8^+^ T cells were evaluated on flow cytometer. The results are the representatives of three independent experiments. (DOCX 93 kb) Additional file 5: Figure S4. CD11c^+^ CD8^+^ T cells are induced by Ag and TLR2 adjuvant therapy. a EG7 tumorbearing mice were treated with OVA or OVA + MALP2s at day 5 and 12 after tumor implantation. Tumor volume was measured every 2 to 3 days. b DLN and tumors were harvested at day 18 and the proportions of OVA-specific CD8^+^ T cells and CD11c^+^ CD8^+^ T cells were evaluated on flow cytometer. Error bars show ± SEM; n = 3 to 5 per group. Student’s *t*-test was performed to analyze statistical significance. * *p* < 0.05. (DOCX 107 kb) Additional file 6: Figure S5. Intratumor CD11c^+^ CD8^+^ T cells show high expression of the cytotoxic genes in mice bearing WT1-C1498 tumor. WT1-C1498 cells were implanted to wild-type mice, and PBS or Poly(I:C) was administered around the tumor at day 5 after tumor implantation. CD11c^-^ and CD11c^+^ CD8^+^ T cells were isolated from tumor tissues by FACS sorting on day 12. The gene expression levels were measured by quantitative PCR. Error bars show ± SD. (DOCX 64.1 kb) Additional file 7: Figure S6. Expansion of CD11c^+^ CD8^+^ T cells in CMV Ag-treated PBMCs isolated from additional donors. PBMCs isolated from three donors were incubated with or without CMV pp65 protein as in Figure 7 in Poly(I:C)-free conditions. PBMCs from donor-2 and donor-3 were incubated for 10 days. PBMCs from donor-4 were incubated for 8 days. Then, the proportions of CMV pp65 tetramer^+^ CD8^+^ T cells (upper panels) and CD11c^+^ CD8^+^ T cells (lower panels) were evaluated on flow cytometer. (DOCX 174 kb)

## Abbreviations

Ag: Antigen
CFSE: Carboxyfluorescein succinimidyl ester
CMV: Cytomegalovirus
CTL: Cytotoxic T lymphocyte
DC: Dendritic cell
DLN: Draining lymph node
IDO: Indoleamine-pyrrole 2,3-dioxygenase
IFN: Interferon
IL: Interleikin
MALP2s: Macrophage-activating lipoprotein-2 s
MAVS: Mitochondrial antiviral signal
MDA5: Melanoma differentiation-associated gene 5
OVA: Ovalbumin
PBMC: Peripheral blood mononuclear cell
PD-L1: Programmed death-ligand 1 (PD-L1)
RRR: Pattern-recognition receptors
TAA: Tumor-associated antigens
TICAM-1: TIR domain-containing adapter molecule 1
TLR: Toll-like receptor
WT1: Wilms’s tumor 1
