# Supplementary material for: Tumoricidal efficacy coincides with CD11c up-regulation in antigen-specific CD8+ T cells during vaccine immunotherapy
Source: J Exp Clin Cancer Res. 2016 Sep 13;35(1):143. doi: 10.1186/s13046-016-0416-x (PMC5020536; doi:10.1186/s13046-016-0416-x)
Supplement: Additional file 1: Table S1. — Primer sequences used for real-time RT-PCR. (DOCX 20.7 kb) [file 13046_2016_416_MOESM1_ESM.docx]

**Supplemental Table 1.** Primer sequences used for real-time RT-PCR.

Primers for samples isolated by TRIzol

| **Gene** | **Primer sequences** | |
| --- | --- | --- |
|  | **Forward** | **Reverse** |
| *Gapdh* | 5’-GCCTGGAGAAACCTGCCA-3’ | 5’-CCCTCAGATGCCTGCTTCA-3’ |
| *Cxcr3* | 5’-GCAAGTTCCCAACCACAAGTG-3’ | 5’-CAAAGTCCGAGGCATCTAGCA-3’ |
| *Gzmb* | 5’-TCCTGCTACTGCTGACCTTGTC-3’ | 5’-ATGATCTCCCCTGCCTTTGTC-3’ |
| *Ifng* | 5’-GATATCTGGAGGAACTGGCAAAAG-3’ | 5’-AGAGATAATCTGGCTCTGCAGGAT-3’ |
| *Il10* | 5’-GGCGCTGTCATCGATTTCTC-3’ | 5’-TGCTCCACTGCCTTGCTCTTA-3’ |
| *Il12rb1* | 5’-AGTTGCGAATGGACTGGAATG-3’ | 5’-GTCACCCAAGGTCCAATTCGT-3’ |
| *Il12rb2* | 5’-AAGTCCCCAAGGAAATGAAAGG-3’ | 5’-GTGATGATAGCGATGCAAATGC-3’ |
| *Pdcd1* | 5’-TGGCTTCTAGAGGTCCCCAAT-3’ | 5’-TGGCATTTGCTCCCTCTGA-3’ |
| *Prf1* | 5’-CAAGGTAGCCAATTTTGCAGC-3’ | 5’-GGCGAAAACTGTACATGCGAC-3’ |
| *Cd127* | 5’-GCCAAAAACGAGTCTGAATGTG-3’ | 5’-TTCAACGCCTTTCACCTCATG-3’ |
| *Klrg1* | 5’-GGCACCTCAAGTCCAAGATGA-3’ | 5’-ATTGCAAAGCGGGAAAGATG-3’ |
| *Cd103* | 5’-CCGTGGCTCCAGAAAAAAAG-3’ | 5’-ATGCATCACCCACCCCAAT-3’ |
| *Tgfb2* | 5’-TCCCGAATAAAAGCGAAGAGC-3’ | 5’-GGTGCCATCAATACCTGCAAA-3’ |
| *Cd122* | 5’-TCCCAGAGTCCCAGTCACTGA-3’ | 5’-CCTACGCCAACCCTTCTCTTC-3’ |
| *Tim3* | 5’-TTGACCCTGGCACTTATCATTG-3’ | 5’-TTCCTCAGAGCGAATCCTGACT-3’ |
| *Lag3* | 5’-CTTCCCTGCAGCCTCAAATC-3’ | 5’-TGGCCACTGTCTGGTTGATG-3’ |

Primers for samples isolated by Transcriptome Amplification Kit

| **Gene** | **Primer sequences** | |
| --- | --- | --- |
|  | **Forward** | **Reverse** |
| *Gapdh* | 5’-GCCTGGAGAAACCTGCCA-3’ | 5’-CCCTCAGATGCCTGCTTCA-3’ |
| *Gzmb* | 5’-CTGGCTTCATGTCCATTCACA-3’ | 5’-GCAGAAGAGGTGTTCCATTGG-3’ |
| *Ifng* | 5’-TGCTGATGGGAGGAGATGTCT-3’ | 5’-TGCTGTCTGGCCTGCTGTTA-3’ |
| *Pdcd1* | 5’-CTGTGCCTGGAAATGGAGAGA-3’ | 5’-TGCCTCACAGGGCCATTC-3’ |
| *Prf1* | 5’- CTCTGGTTTCCATGAGGTGACA-3’ | 5’-GGCAGACACTTGGCATGGTA-3’ |
