# Supplementary material for: Tumoricidal efficacy coincides with CD11c up-regulation in antigen-specific CD8+ T cells during vaccine immunotherapy
Source: J Exp Clin Cancer Res. 2016 Sep 13;35(1):143. doi: 10.1186/s13046-016-0416-x (PMC5020536; doi:10.1186/s13046-016-0416-x)
Supplement: Additional file 2: Figure S1. — CD11c+ CD8+ T cells show Ag + Poly(I:C)-induced effector functions in non-tumor bearing mice. a-b OVA with or without Poly(I:C) was s.c. injected to tumor-unloading mice. Seven days later, spleens and DLN were harvested and the proportions of OVA-specific CD8+ T cells, CD11c+ CD8+ T cells (a) and IFN-g+ CD8+ T cells (b) were evaluated by flow cytometer. Error bars show ± SEM; n = 5 per group. Student’s t-test was performed for statistical significance. * p < 0.05 (a, b). c CD11c- CD8+ T cells and CD11c+ CD8+ T cells were isolated from spleens by flow cytometer sorting at day 7. Isolated cells were cocultured with 51Cr-labeled EG7 or EL4 (E/T = 20) for 4 hours. Then, the cytotoxicity against EG7 and EL4 was measured by 51Cr-release assay. Error bars show ± SEM. (DOCX 128 kb) [file 13046_2016_416_MOESM2_ESM.docx]

**Supplemental Figure 1.** CD11c^+^ CD8^+^ T cells show Ag + Poly(I:C)-induced effector functions in non-tumor bearing mice.

**a-b** OVA with or without Poly(I:C) was s.c. injected to tumor-unloading mice. Seven days later, spleens and DLN were harvested and the proportions of OVA-specific CD8^+^ T cells, CD11c^+^ CD8^+^ T cells (a) and IFN-γ^+^ CD8^+^ T cells (b) were evaluated by flow cytometer. Error bars show ± SEM; n = 5 per group. Student’s *t*-test was performed for statistical significance. * p < 0.05 (a, b). **c** CD11c^-^ CD8^+^ T cells and CD11c^+^ CD8^+^ T cells were isolated from spleens by flow cytometer sorting at day 7. Isolated cells were co-cultured with ^51^Cr-labeled EG7 or EL4 (E/T = 20) for 4 hours. Then, the cytotoxicity against EG7 and EL4 was measured by ^51^Cr-release assay. Error bars show ± SEM.
