# Supplementary material for: Tumoricidal efficacy coincides with CD11c up-regulation in antigen-specific CD8+ T cells during vaccine immunotherapy
Source: J Exp Clin Cancer Res. 2016 Sep 13;35(1):143. doi: 10.1186/s13046-016-0416-x (PMC5020536; doi:10.1186/s13046-016-0416-x)
Supplement: Additional file 3: Figure S2. — Gene expression profile of CD11c+ CD8+ T cells. a-b EG7 tumor-bearing mice were treated with OVA or OVA + Poly(I:C) at day 9. Six days later, spleens were harvested and the proportions of CD62L-, PD-1+, KLRG1+ /CD127- and CD103+ of the CD8+ T cells (a) and TNF-a+ and IL-2+ of the CD8+ T cells (b) were evaluated by flow cytometer. c CD11c- CD8+ and CD11c+ CD8+ T cells were isolated from spleens and tumors by FACS sorting at day 15. The gene expression levels were measured by quantitative PCR. Error bars show ± SEM; n = 3 to 9 per group (a, b) and ± SD (c). Student’s t-test was performed for statistical significance. * p < 0.05. The results are one of the two independent experiments. (DOCX 214 kb) [file 13046_2016_416_MOESM3_ESM.docx]

**Supplemental Figure 2.** Gene expression profile of CD11c^+^ CD8^+^ T cells.

**a-b** EG7 tumor-bearing mice were treated with OVA or OVA + Poly(I:C) at day 9. Six days later, spleens were harvested and the proportions of CD62L^-^, PD-1^+^, KLRG1^+^ /CD127^-^ and CD103^+^ of the CD8^+^ T cells (a) and TNF-α^+^ and IL-2^+^ of the CD8^+^ T cells (b) were evaluated by flow cytometer. **c** CD11c^-^ CD8^+^ and CD11c^+^ CD8^+^ T cells were isolated from spleens and tumors by FACS sorting at day 15. The gene expression levels were measured by quantitative PCR. Error bars show ± SEM; n = 3 to 9 per group (a, b) and ± SD (c). Student’s *t*-test was performed for statistical significance. * p < 0.05. The results are one of the two independent experiments.
