# Supplementary material for: Tumoricidal efficacy coincides with CD11c up-regulation in antigen-specific CD8+ T cells during vaccine immunotherapy
Source: J Exp Clin Cancer Res. 2016 Sep 13;35(1):143. doi: 10.1186/s13046-016-0416-x (PMC5020536; doi:10.1186/s13046-016-0416-x)
Supplement: Additional file 4: Figure S3. — Tumor-infiltrating CD11c+ CD8+ T cells disappear immediately before the complete tumor regression. a EG7 tumor-bearing mice were administered with OVA and Poly(I:C) around tumor at day 5 after tumor implantation. Tumor volume was measured every 2 to 3 days. Tumors with mild (+) and rapid (++) regression profiles (≥ 200 mm3 at day 12 vs. ≤ 200 mm3 at day 12) were used in this study. b 7 days after OVA treatment (on day 12), the proportions of intratumor OVA-specific CD8+ T cells and CD11c+ CD8+ T cells were evaluated on flow cytometer. The results are the representatives of three independent experiments. (DOCX 93 kb) [file 13046_2016_416_MOESM4_ESM.docx]

**Supplemental Figure 3.** Tumor-infiltrating CD11c^+^ CD8^+^ T cells disappear immediately before the complete tumor regression.

**a** EG7 tumor-bearing mice were administered with OVA and Poly(I:C) around tumor at day 5 after tumor implantation. Tumor volume was measured every 2 to 3 days. Tumors with mild (+) and rapid (++) regression profiles (≥ 200 mm^3^ at day 12 vs. ≤ 200 mm^3^ at day 12) were used in this study. **b** 7 days after OVA treatment (on day 12), the proportions of intratumor OVA-specific CD8^+^ T cells and CD11c^+^ CD8^+^ T cells were evaluated on flow cytometer. The results are the representatives of three independent experiments.
