# Supplementary material for: Tumoricidal efficacy coincides with CD11c up-regulation in antigen-specific CD8+ T cells during vaccine immunotherapy
Source: J Exp Clin Cancer Res. 2016 Sep 13;35(1):143. doi: 10.1186/s13046-016-0416-x (PMC5020536; doi:10.1186/s13046-016-0416-x)
Supplement: Additional file 5: Figure S4. — CD11c+ CD8+ T cells are induced by Ag and TLR2 adjuvant therapy. a EG7 tumorbearing mice were treated with OVA or OVA + MALP2s at day 5 and 12 after tumor implantation. Tumor volume was measured every 2 to 3 days. b DLN and tumors were harvested at day 18 and the proportions of OVA-specific CD8+ T cells and CD11c+ CD8+ T cells were evaluated on flow cytometer. Error bars show ± SEM; n = 3 to 5 per group. Student’s t-test was performed to analyze statistical significance. * p < 0.05. (DOCX 107 kb) [file 13046_2016_416_MOESM5_ESM.docx]

**Supplemental Figure 4.** CD11c^+^ CD8^+^ T cells are induced by Ag and TLR2 adjuvant therapy.

**a** EG7 tumor-bearing mice were treated with OVA or OVA + MALP2s at day 5 and 12 after tumor implantation. Tumor volume was measured every 2 to 3 days. **b** DLN and tumors were harvested at day 18 and the proportions of OVA-specific CD8^+^ T cells and CD11c^+^ CD8^+^ T cells were evaluated on flow cytometer. Error bars show ± SEM; n = 3 to 5 per group. Student’s t-test was performed to analyze statistical significance. * p < 0.05.
