# Supplementary material for: Tumoricidal efficacy coincides with CD11c up-regulation in antigen-specific CD8+ T cells during vaccine immunotherapy
Source: J Exp Clin Cancer Res. 2016 Sep 13;35(1):143. doi: 10.1186/s13046-016-0416-x (PMC5020536; doi:10.1186/s13046-016-0416-x)
Supplement: Additional file 6: Figure S5. — Intratumor CD11c+ CD8+ T cells show high expression of the cytotoxic genes in mice bearing WT1-C1498 tumor. WT1-C1498 cells were implanted to wild-type mice, and PBS or Poly(I:C) was administered around the tumor at day 5 after tumor implantation. CD11c- and CD11c+ CD8+ T cells were isolated from tumor tissues by FACS sorting on day 12. The gene expression levels were measured by quantitative PCR. Error bars show ± SD. (DOCX 64.1 kb) [file 13046_2016_416_MOESM6_ESM.docx]

**Supplemental Figure 5.** Intratumor CD11c^+^ CD8^+^ T cells show high expression of the cytotoxic genes in mice bearing WT1-C1498 tumor.

WT1-C1498 cells were implanted to wild-type mice, and PBS or Poly(I:C) was administered around the tumor at day 5 after tumor implantation. CD11c^-^ and CD11c^+^ CD8^+^ T cells were isolated from tumor tissues by FACS sorting on day 12. The gene expression levels were measured by quantitative PCR. Error bars show ± SD.
