# Supplementary material for: Tumoricidal efficacy coincides with CD11c up-regulation in antigen-specific CD8+ T cells during vaccine immunotherapy
Source: J Exp Clin Cancer Res. 2016 Sep 13;35(1):143. doi: 10.1186/s13046-016-0416-x (PMC5020536; doi:10.1186/s13046-016-0416-x)
Supplement: Additional file 7: Figure S6. — Expansion of CD11c+ CD8+ T cells in CMV Ag-treated PBMCs isolated from additional donors. PBMCs isolated from three donors were incubated with or without CMV pp65 protein as in Figure 7 in Poly(I:C)-free conditions. PBMCs from donor-2 and donor-3 were incubated for 10 days. PBMCs from donor-4 were incubated for 8 days. Then, the proportions of CMV pp65 tetramer+ CD8+ T cells (upper panels) and CD11c+ CD8+ T cells (lower panels) were evaluated on flow cytometer. (DOCX 174 kb) [file 13046_2016_416_MOESM7_ESM.docx]

**Supplemental Figure 6.** Expansion of CD11c^+^ CD8^+^ T cells in CMV Ag-treated PBMCs isolated from additional donors.

PBMCs isolated from three donors were incubated with or without CMV pp65 protein as in Figure 7 in Poly(I:C)-free conditions. PBMCs from donor-2 and donor-3 were incubated for 10 days. PBMCs from donor-4 were incubated for 8 days. Then, the proportions of CMV pp65 tetramer^+^ CD8^+^ T cells (upper panels) and CD11c^+^ CD8^+^ T cells (lower panels) were evaluated on flow cytometer.
